# Crystal structure of (*E*)-2-{[(4-anilinophen­yl)imino]­meth­yl}phenol

**DOI:** 10.1107/S2056989014026309

**Published:** 2015-01-01

**Authors:** Md. Serajul Haque Faizi, Turganbay S. Iskenderov, Natalia O. Sharkina

**Affiliations:** a Department of Chemistry, Indian Institute of Technology Kanpur, Kanpur, UP 208 016, India; b National Taras Shevchenko University, Department of Chemistry, Volodymyrska str. 64, 01601 Kyiv, Ukraine

**Keywords:** crystal structure, *N*-phenyl-*p*-phenyl­enedi­amine, salicyladehyde, PAIMP, Schiff base, hydrogen bonding

## Abstract

The title compound, C_19_H_16_N_2_O, crystallized with two independent mol­ecules (*A* and *B*) in the asymmetric unit. There is an intra­molecular O—H⋯N hydrogen bond in each mol­ecule with the phenol ring being inclined to the central benzene ring by 4.93 (14) and 7.12 (14)° in mol­ecules *A* and *B*, respectively. The conformation of the two mol­ecules differs essentially in the orientation of the terminal amino­phenyl ring with respect to the central benzene ring; this dihedral angle is 50.51 (4)° in mol­ecule *A* and 54.61 (14)° in mol­ecule *B*. The two outer aromatic rings are inclined to one another by 51.39 (14) and 49.88 (14)° in mol­ecules *A* and *B*, respectively. In the crystal, mol­ecules are connected by N—H⋯O hydrogen bonds generating –*A*-*B*–*A*–*B*– zigzag chains extending along [010]. The chains are linked *via* C—H⋯π inter­actions involving neighbouring *A* mol­ecules, forming slabs lying parallel to (100).

## Abstract 2

The title compound crystallized with two independent mol­ecules (*A* and *B*) in the asymmetric unit, which differ essentially in the orientation of the terminal amino­phenyl ring with respect to the central benzene ring. In the crystal, mol­ecules are linked *via* N—H⋯O hydrogen bonds forming –*A*-*B*–*A*–*B*– zigzag chains propagating along [010].

## Chemical context   

Schiff bases often exhibit various biological activities and in many cases have been shown to have anti­bacterial, anti­cancer, anti-inflammatory and anti­toxic properties (Lozier *et al.*, 1975 ▸). They are used as anion sensors (Dalapati *et al.*, 2011 ▸), as non-linear optics compounds (Sun *et al.*, 2012 ▸) and as versatile polynuclear ligands for multinuclear magnetic exchange clusters (Moroz *et al.*, 2012 ▸). Schiff bases have also been used to prepare metal complexes (Faizi & Sen, 2014 ▸; Faizi & Hussain, 2014 ▸; Penkova *et al.*, 2010 ▸). We report herein on the crystal structure of the title compound synthesized by the condensation reaction of salicyladehyde and *N*-phenyl-*p*-phenyl­enedi­amine.
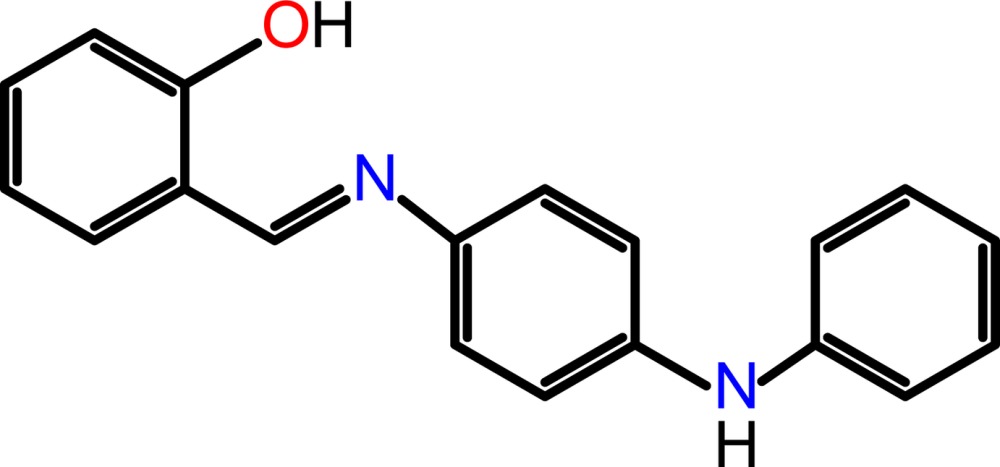


## Structural commentary   

**Figure 1:**
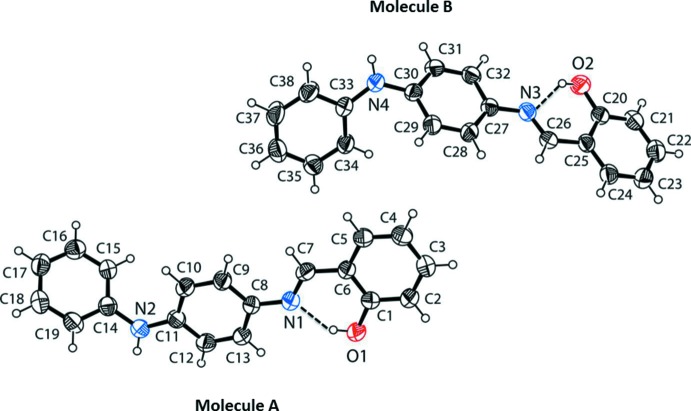
The mol­ecular structure of the two independent mol­ecules (*A* and *B*) of the title compound, with the atom labelling. Displacement ellipsoids are drawn at the 40% probability level. Intra­molecular O—H⋯N hydrogen bonds are shown as dashed lines (see Table 1 ▸ for details).

The title compound crystallized with two independent mol­ecules (*A* and *B*) in the asymmetric unit (Fig. 1 ▸). There is an intra­molecular O—H⋯N hydrogen bond in each mol­ecule, which is a common feature in related imine-phenol compounds and it stabilizes the mol­ecular structure (Table 1 ▸ and Fig. 1 ▸). The imine group displays a torsion angle C6—C7—N1—C8 = 178.8 (2)° in mol­ecule *A* and C25—C26—N3—C27 = 178.5 (2)° in mol­ecule *B*. In mol­ecules *A* and *B* the phenol rings (C1–C6 and C20–C25) are inclined to the central benzene rings (C8–C13 and C27–C32) by 4.93 (14) and 7.12 (14)°, respectively.

The conformation of the two mol­ecules differs essentially in the orientation of the terminal amino­phenyl rings (C14–C19 and C33–C38) with respect to the central benzene rings (C8–C13 and C27–C32); this dihedral angle is 50.51 (4)° in mol­ecule *A* and 54.61 (14)° in mol­ecule *B*. The two outer aromatic rings (C1–C6 and C14–C19 in *A*, and C20–C25 and C33–C38 in *B*) are inclined to one another by 51.39 (14) and 49.88 (14)° in mol­ecules *A* and *B*, respectively. The C—N, C N and C—C bond lengths are normal and close to the values observed in related structures (Sliva *et al.*, 1997 ▸; Petrusenko *et al.*, 1997 ▸).

## Supra­molecular features   

**Figure 2:**
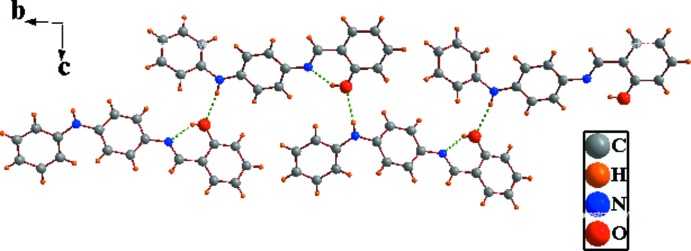
A view of the –*A*-*B*–*A*–*B*– zigzag hydrogen-bonded chain in the crystal of the title compound, extending along the *b* axis (hydrogen bonds are shown as dashed lines; see Table 1 ▸ for details).

**Figure 3:**
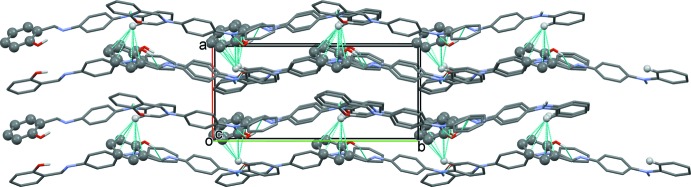
A view along the *c* axis of the crystal packing of the title compound. The hydrogen bonds and C—H⋯π inter­actions are shown as dashed lines (see Table 1 ▸ for details; for the latter inter­actions the atoms involved are shown as light and dark grey balls).

In the crystal, mol­ecules are connected by N—H⋯O hydrogen bonds, generating –*A*-*B*–*A*–*B*– zigzag chains extending along [010]; Table 1 ▸ and Fig. 2 ▸. The chains are linked *via* C—H⋯π inter­actions involving neighbouring *A* mol­ecules, forming slabs lying parallel to (100); see Table 1 ▸ and Fig. 3 ▸.

## Database survey   

There are very few examples of similar compounds in the literature although some metal complexes of similar ligands have been reported on (Xie *et al.*, 2013 ▸; Safin *et al.*, 2012 ▸). A search of the Cambridge Structural Database (Version 5.35, May 2014; Groom & Allen, 2014 ▸) revealed the structure of one very similar compound, *viz. N*-[(*E*)-4-chloro­benzyl­idene]-*N*′-phenyl­benzene-1,4-di­amine (II) (Nor Hashim *et al.*, 2010 ▸), in which the 2-phenol ring in the title compound is replaced by a 4-chloro­benzene ring. In (II), the central six-membered ring makes a dihedral angle of 12.26 (10)° with the 4-chloro­phenyl ring. The same dihedral angle is smaller in the title compound, 4.93 (14)° in mol­ecule *A* and 7.12 (14)° in mol­ecule *B*, owing to the presence of the intra­molecular O—H⋯N hydrogen bond. The outer phenyl ring is inclined to the central six-membered ring by 44.18 (11)° in (II), compared to 50.51 (4) and 54.61 (14)° in mol­ecules *A* and *B*, respectively, of the title compound.

## Synthesis and crystallization   

100 mg (1 mmol) of *N*-phenyl-*p*-phenyl­enedi­amine were dissolved in 10 ml of absolute ethanol. To this solution, 66 mg (1 mmol) of salicyladehyde in 5 ml of absolute ethanol was added dropwise with stirring. The mixture was stirred for 10 min, two drops of glacial acetic acid were then added and the mixture was further refluxed for 2 h. The resulting reddish yellow precipitate was recovered by filtration, washed several times with a small portions of EtOH and then with diethyl ether to give 120 mg (75%) of the title compound. Crystals suitable for X-ray analysis was obtained within 3 days by slow evaporation of a solution in methanol.

## Refinement   

Crystal data, data collection and structure refinement details are summarized in Table 2 ▸. The N—H and O—H H atoms were located from a difference Fourier map and constrained to ride on their parent atoms, with N—H = 0.86 and O—H = 0.82 Å and with *U*_iso_(H) = 1.2*U*_eq_(N) and = 1.5*U*_eq_(O). All C-bound H atoms were positioned geometrically and refined using a riding model with C—H = 0.93 Å and with *U*_iso_(H) = 1.2*U*_eq_(C).

## Supplementary Material

Crystal structure: contains datablock(s) global, I. DOI: 10.1107/S2056989014026309/su5028sup1.cif

Structure factors: contains datablock(s) I. DOI: 10.1107/S2056989014026309/su5028Isup2.hkl

Supporting information file. DOI: 10.1107/S2056989014026309/su5028Isup3.cml

CCDC reference: 1036844

Additional supporting information:  crystallographic information; 3D view; checkCIF report

## Figures and Tables

**Table 1:** Hydrogen-bond geometry (, ) *Cg*1 is the centroid of ring C1C6 in molecule *A*.

*D*H*A*	*D*H	H*A*	*D* *A*	*D*H*A*
O1H4*A*N1	0.82	1.86	2.568(3)	144
O2H3*A*N3	0.82	1.82	2.550(3)	148
N2H1*A*O2^i^	0.86	2.29	3.006(4)	141
N4H2*A*O1^ii^	0.86	2.33	3.179(4)	168
C15H13*Cg*1^iii^	0.93	2.92	3.581(4)	129

Symmetry codes: (i) 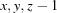; (ii) 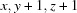; (iii) 
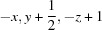
.

**Table 2:** Experimental details

Crystal data	
Chemical formula	C_19_H_16_N_2_O
*M*_r_	288.34
Crystal system, space group	Monoclinic, *P*2_1_
Temperature (K)	100
*a*, *b*, *c* ()	7.704(6), 16.706(12), 11.617(9)
()	93.880(14)
*V* (^3^)	1492(2)
*Z*	4
Radiation type	Mo *K*
(mm^1^)	0.08
Crystal size (mm)	0.20 0.15 0.12

Data collection	
Diffractometer	Bruker SMART APEX CCD
Absorption correction	Multi-scan (*SADABS*; Sheldrick, 2004 ▸)
*T*_min_, *T*_max_	0.986, 0.990
No. of measured, independent and observed [*I* > 2(*I*)] reflections	8013, 5245, 4091
*R*_int_	0.026
(sin /)_max_ (^1^)	0.606

Refinement	
*R*[*F* ^2^ > 2(*F* ^2^)], *wR*(*F* ^2^), *S*	0.045, 0.127, 0.97
No. of reflections	5242
No. of parameters	397
No. of restraints	1
H-atom treatment	H-atom parameters constrained
_max_, _min_ (e ^3^)	0.12, 0.15

Computer programs: *SMART* and *SAINT* (Bruker, 2003 ▸), *SIR97* (Altomare *et al.*, 1999 ▸), *SHELXL97* (Sheldrick, 2008 ▸), *DIAMOND* (Brandenberg Putz, 2006 ▸), *Mercury* (Macrae *et al.*, 2008 ▸) and *PLATON* (Spek, 2009 ▸).

## supplementary crystallographic information

### Crystal data

**Table:**

C_19_H_16_N_2_O	*F*(000) = 608
*M**_r_* = 288.34	*D*_x_ = 1.284 Mg m^−^^3^
Monoclinic, *P*2_1_	Melting point: 280 K
Hall symbol: P 2yb	Mo *K*α radiation, λ = 0.71073 Å
*a* = 7.704 (6) Å	Cell parameters from 2553 reflections
*b* = 16.706 (12) Å	θ = 2.7–23.7°
*c* = 11.617 (9) Å	µ = 0.08 mm^−^^1^
β = 93.880 (14)°	*T* = 100 K
*V* = 1492 (2) Å^3^	Needle, dark yellow
*Z* = 4	0.20 × 0.15 × 0.12 mm

### Data collection

**Table:**

Bruker SMART APEX CCD diffractometer	5245 independent reflections
Radiation source: fine-focus sealed tube	4091 reflections with *I* > 2σ(*I*)
Graphite monochromator	*R*_int_ = 0.026
ω scans	θ_max_ = 25.5°, θ_min_ = 1.8°
Absorption correction: multi-scan (*SADABS*; Sheldrick, 2004)	*h* = −9→7
*T*_min_ = 0.986, *T*_max_ = 0.990	*k* = −20→19
8013 measured reflections	*l* = −14→13

### Refinement

**Table:**

Refinement on *F*^2^	Primary atom site location: structure-invariant direct methods
Least-squares matrix: full	Secondary atom site location: difference Fourier map
*R*[*F*^2^ > 2σ(*F*^2^)] = 0.045	Hydrogen site location: inferred from neighbouring sites
*wR*(*F*^2^) = 0.127	H-atom parameters constrained
*S* = 0.97	*w* = 1/[σ^2^(*F*_o_^2^) + (0.0711*P*)^2^ + 0.0812*P*] where *P* = (*F*_o_^2^ + 2*F*_c_^2^)/3
5242 reflections	(Δ/σ)_max_ < 0.001
397 parameters	Δρ_max_ = 0.12 e Å^−^^3^
1 restraint	Δρ_min_ = −0.15 e Å^−^^3^

### Special details

**Table:**

Geometry. All e.s.d.'s (except the e.s.d. in the dihedral angle between two l.s. planes) are estimated using the full covariance matrix. The cell e.s.d.'s are taken into account individually in the estimation of e.s.d.'s in distances, angles and torsion angles; correlations between e.s.d.'s in cell parameters are only used when they are defined by crystal symmetry. An approximate (isotropic) treatment of cell e.s.d.'s is used for estimating e.s.d.'s involving l.s. planes.
Refinement. Refinement of *F*^2^ against ALL reflections. The weighted *R*-factor *wR* and goodness of fit *S* are based on *F*^2^, conventional *R*-factors *R* are based on *F*, with *F* set to zero for negative *F*^2^. The threshold expression of *F*^2^ > σ(*F*^2^) is used only for calculating *R*-factors(gt) *etc*. and is not relevant to the choice of reflections for refinement. *R*-factors based on *F*^2^ are statistically about twice as large as those based on *F*, and *R*- factors based on ALL data will be even larger.

### Fractional atomic coordinates and isotropic or equivalent isotropic displacement parameters (Å^2^)

**Table:**

	*x*	*y*	*z*	*U*_iso_*/*U*_eq_	
C15	0.2606 (3)	0.66796 (17)	0.4413 (2)	0.0599 (7)	
H13	0.2251	0.6192	0.4706	0.072*	
C16	0.2437 (4)	0.73672 (18)	0.5044 (3)	0.0642 (7)	
H14	0.1982	0.7337	0.5764	0.077*	
C17	0.2923 (4)	0.80966 (19)	0.4637 (3)	0.0763 (9)	
H15	0.2800	0.8559	0.5070	0.092*	
C18	0.3596 (5)	0.8129 (2)	0.3573 (3)	0.0834 (10)	
H16	0.3913	0.8621	0.3276	0.100*	
C19	0.3807 (4)	0.74423 (19)	0.2938 (3)	0.0715 (8)	
H17	0.4296	0.7474	0.2229	0.086*	
C14	0.3301 (3)	0.67070 (16)	0.3346 (2)	0.0544 (6)	
C11	0.3031 (3)	0.52520 (15)	0.2888 (2)	0.0538 (6)	
C12	0.2085 (4)	0.47990 (17)	0.2069 (2)	0.0605 (7)	
H20	0.1685	0.5035	0.1377	0.073*	
C13	0.1725 (4)	0.40068 (17)	0.2262 (2)	0.0605 (7)	
H21	0.1083	0.3717	0.1698	0.073*	
C8	0.2303 (3)	0.36316 (15)	0.3284 (2)	0.0515 (6)	
C9	0.3247 (4)	0.40894 (15)	0.4112 (2)	0.0595 (7)	
H23	0.3630	0.3856	0.4810	0.071*	
C10	0.3620 (4)	0.48772 (16)	0.3914 (2)	0.0602 (7)	
H24	0.4275	0.5167	0.4472	0.072*	
C1	0.0687 (3)	0.12055 (16)	0.3414 (2)	0.0569 (7)	
C2	0.0062 (4)	0.04379 (18)	0.3517 (3)	0.0647 (7)	
H26	−0.0649	0.0215	0.2922	0.078*	
C3	0.0489 (4)	−0.00004 (18)	0.4503 (3)	0.0722 (8)	
H27	0.0082	−0.0522	0.4563	0.087*	
C4	0.1521 (4)	0.03320 (18)	0.5403 (3)	0.0696 (8)	
H28	0.1804	0.0036	0.6067	0.083*	
C5	0.2121 (4)	0.10975 (17)	0.5308 (3)	0.0621 (7)	
H29	0.2825	0.1315	0.5911	0.075*	
C6	0.1703 (3)	0.15580 (16)	0.4335 (2)	0.0525 (6)	
C7	0.2253 (3)	0.23864 (16)	0.4276 (2)	0.0542 (6)	
H37	0.2917	0.2607	0.4895	0.065*	
N2	0.3442 (3)	0.60413 (13)	0.2630 (2)	0.0663 (7)	
H1A	0.3817	0.6130	0.1961	0.080*	
N1	0.1843 (3)	0.28196 (13)	0.3392 (2)	0.0560 (6)	
O1	0.0278 (3)	0.16115 (13)	0.24309 (16)	0.0714 (5)	
H4A	0.0827	0.2032	0.2434	0.107*	
C33	0.1682 (3)	1.17385 (17)	0.9328 (2)	0.0590 (7)	
C38	0.2378 (4)	1.24246 (19)	0.9834 (3)	0.0693 (8)	
H2	0.2798	1.2416	1.0603	0.083*	
C37	0.2458 (4)	1.31204 (19)	0.9210 (3)	0.0762 (9)	
H3	0.2929	1.3580	0.9560	0.091*	
C36	0.1842 (4)	1.31411 (19)	0.8067 (3)	0.0707 (8)	
H4	0.1910	1.3609	0.7639	0.085*	
C35	0.1130 (4)	1.24640 (18)	0.7571 (3)	0.0663 (7)	
H5	0.0697	1.2476	0.6804	0.080*	
C34	0.1046 (4)	1.17697 (18)	0.8187 (2)	0.0655 (7)	
H6	0.0557	1.1315	0.7836	0.079*	
C30	0.1879 (4)	1.02639 (16)	0.9723 (2)	0.0565 (7)	
C29	0.2807 (4)	1.00490 (16)	0.8772 (2)	0.0592 (7)	
H8	0.3174	1.0442	0.8276	0.071*	
C28	0.3171 (4)	0.92596 (16)	0.8573 (2)	0.0593 (7)	
H9	0.3780	0.9124	0.7936	0.071*	
C27	0.2653 (3)	0.86564 (15)	0.9300 (2)	0.0532 (6)	
C32	0.1747 (4)	0.88786 (17)	1.0241 (2)	0.0603 (7)	
H11	0.1397	0.8486	1.0743	0.072*	
C31	0.1356 (4)	0.96604 (17)	1.0447 (2)	0.0633 (7)	
H12	0.0733	0.9791	1.1079	0.076*	
C25	0.3974 (3)	0.66532 (15)	0.8267 (2)	0.0511 (6)	
C24	0.4721 (4)	0.63136 (18)	0.7335 (2)	0.0616 (7)	
H32	0.5007	0.6639	0.6727	0.074*	
C23	0.5049 (4)	0.55121 (18)	0.7285 (3)	0.0651 (7)	
H33	0.5572	0.5298	0.6656	0.078*	
C22	0.4601 (4)	0.50243 (18)	0.8170 (3)	0.0640 (7)	
H34	0.4804	0.4476	0.8134	0.077*	
C21	0.3851 (4)	0.53439 (17)	0.9110 (3)	0.0643 (7)	
H35	0.3554	0.5010	0.9706	0.077*	
C20	0.3537 (4)	0.61552 (16)	0.9176 (2)	0.0570 (7)	
C26	0.3651 (4)	0.75097 (17)	0.8317 (2)	0.0577 (7)	
H38	0.3947	0.7833	0.7709	0.069*	
N4	0.1551 (4)	1.10499 (14)	1.0013 (2)	0.0729 (7)	
H2A	0.1231	1.1129	1.0699	0.088*	
N3	0.2966 (3)	0.78277 (13)	0.9181 (2)	0.0577 (6)	
O2	0.2820 (3)	0.64507 (13)	1.01166 (17)	0.0804 (6)	
H3A	0.2716	0.6938	1.0058	0.121*	

### Atomic displacement parameters (Å^2^)

**Table:**

	*U*^11^	*U*^22^	*U*^33^	*U*^12^	*U*^13^	*U*^23^
C15	0.0657 (16)	0.0499 (15)	0.0653 (17)	−0.0044 (13)	0.0135 (13)	0.0061 (14)
C16	0.0660 (18)	0.0598 (18)	0.0674 (17)	−0.0004 (14)	0.0095 (14)	−0.0059 (15)
C17	0.086 (2)	0.0538 (19)	0.088 (2)	−0.0044 (16)	−0.0051 (18)	−0.0101 (17)
C18	0.102 (3)	0.0523 (19)	0.095 (3)	−0.0241 (18)	−0.003 (2)	0.0120 (18)
C19	0.083 (2)	0.0619 (19)	0.0699 (19)	−0.0170 (16)	0.0064 (15)	0.0121 (17)
C14	0.0537 (14)	0.0489 (15)	0.0603 (16)	−0.0029 (12)	0.0026 (12)	0.0069 (13)
C11	0.0606 (16)	0.0503 (15)	0.0518 (15)	0.0064 (12)	0.0117 (12)	0.0039 (12)
C12	0.0727 (18)	0.0622 (18)	0.0466 (15)	0.0055 (14)	0.0038 (13)	0.0020 (13)
C13	0.0654 (17)	0.0652 (19)	0.0504 (16)	0.0026 (14)	0.0005 (13)	−0.0083 (13)
C8	0.0530 (14)	0.0499 (15)	0.0516 (15)	0.0028 (12)	0.0029 (11)	0.0000 (12)
C9	0.0687 (18)	0.0539 (17)	0.0547 (16)	0.0058 (13)	−0.0049 (13)	0.0033 (13)
C10	0.0702 (18)	0.0488 (16)	0.0606 (17)	0.0005 (13)	−0.0022 (13)	−0.0007 (13)
C1	0.0594 (16)	0.0568 (17)	0.0555 (16)	0.0032 (13)	0.0124 (13)	−0.0025 (14)
C2	0.0672 (17)	0.0598 (18)	0.0680 (19)	−0.0055 (14)	0.0120 (14)	−0.0123 (15)
C3	0.077 (2)	0.0545 (17)	0.088 (2)	−0.0032 (15)	0.0223 (17)	−0.0045 (17)
C4	0.080 (2)	0.0582 (18)	0.0706 (19)	0.0098 (16)	0.0088 (16)	0.0067 (15)
C5	0.0650 (17)	0.0576 (17)	0.0636 (18)	0.0059 (14)	0.0027 (14)	−0.0027 (14)
C6	0.0541 (14)	0.0490 (15)	0.0555 (15)	0.0042 (12)	0.0112 (11)	−0.0037 (12)
C7	0.0603 (16)	0.0526 (16)	0.0504 (15)	0.0029 (13)	0.0083 (12)	−0.0037 (13)
N2	0.0920 (18)	0.0493 (14)	0.0597 (14)	0.0024 (13)	0.0212 (13)	0.0051 (11)
N1	0.0609 (13)	0.0488 (13)	0.0589 (14)	0.0015 (10)	0.0092 (11)	−0.0036 (11)
O1	0.0855 (13)	0.0679 (13)	0.0599 (11)	−0.0105 (11)	−0.0026 (9)	−0.0010 (10)
C33	0.0608 (16)	0.0555 (17)	0.0611 (17)	0.0088 (14)	0.0070 (13)	−0.0025 (14)
C38	0.0741 (19)	0.0608 (18)	0.0713 (19)	0.0029 (16)	−0.0063 (15)	−0.0145 (17)
C37	0.087 (2)	0.0529 (19)	0.089 (2)	−0.0051 (16)	0.0059 (18)	−0.0099 (17)
C36	0.077 (2)	0.0549 (18)	0.082 (2)	0.0044 (15)	0.0180 (16)	0.0056 (16)
C35	0.0669 (18)	0.0671 (19)	0.0648 (17)	0.0043 (15)	0.0047 (14)	0.0028 (16)
C34	0.0784 (19)	0.0552 (17)	0.0628 (17)	−0.0073 (15)	0.0030 (14)	−0.0036 (14)
C30	0.0624 (17)	0.0513 (16)	0.0552 (15)	0.0023 (13)	−0.0003 (13)	−0.0036 (13)
C29	0.0659 (17)	0.0544 (17)	0.0583 (16)	−0.0025 (13)	0.0108 (13)	0.0049 (13)
C28	0.0635 (17)	0.0556 (17)	0.0604 (16)	−0.0003 (13)	0.0147 (13)	0.0012 (13)
C27	0.0584 (15)	0.0493 (16)	0.0521 (15)	−0.0045 (12)	0.0058 (12)	0.0017 (12)
C32	0.0705 (18)	0.0588 (18)	0.0525 (15)	−0.0069 (14)	0.0112 (13)	0.0045 (13)
C31	0.0710 (18)	0.0634 (18)	0.0570 (16)	0.0022 (14)	0.0145 (14)	0.0009 (14)
C25	0.0513 (14)	0.0506 (16)	0.0515 (15)	−0.0019 (12)	0.0025 (11)	0.0033 (13)
C24	0.0654 (17)	0.0656 (19)	0.0551 (16)	−0.0014 (14)	0.0135 (13)	0.0012 (13)
C23	0.0669 (17)	0.067 (2)	0.0625 (17)	0.0038 (14)	0.0140 (14)	−0.0039 (15)
C22	0.0665 (18)	0.0533 (17)	0.0722 (19)	−0.0006 (14)	0.0046 (14)	−0.0061 (15)
C21	0.081 (2)	0.0512 (17)	0.0612 (18)	−0.0003 (14)	0.0117 (15)	0.0074 (13)
C20	0.0666 (17)	0.0566 (17)	0.0482 (15)	−0.0006 (13)	0.0066 (13)	−0.0008 (13)
C26	0.0631 (16)	0.0589 (18)	0.0516 (15)	−0.0035 (13)	0.0070 (13)	0.0083 (14)
N4	0.105 (2)	0.0584 (16)	0.0569 (14)	0.0095 (14)	0.0161 (13)	−0.0026 (12)
N3	0.0646 (14)	0.0497 (14)	0.0591 (14)	−0.0009 (11)	0.0060 (11)	0.0018 (11)
O2	0.1272 (18)	0.0600 (13)	0.0575 (12)	0.0089 (12)	0.0318 (12)	0.0068 (9)

### Geometric parameters (Å, º)

**Table:**

C15—C16	1.373 (4)	C33—C34	1.383 (4)
C15—C14	1.384 (4)	C33—C38	1.380 (4)
C15—H13	0.9300	C33—N4	1.406 (4)
C16—C17	1.368 (4)	C38—C37	1.373 (5)
C16—H14	0.9300	C38—H2	0.9300
C17—C18	1.374 (5)	C37—C36	1.381 (5)
C17—H15	0.9300	C37—H3	0.9300
C18—C19	1.380 (5)	C36—C35	1.367 (4)
C18—H16	0.9300	C36—H4	0.9300
C19—C14	1.382 (4)	C35—C34	1.367 (4)
C19—H17	0.9300	C35—H5	0.9300
C14—N2	1.397 (4)	C34—H6	0.9300
C11—C12	1.384 (4)	C30—N4	1.383 (4)
C11—N2	1.393 (3)	C30—C31	1.390 (4)
C11—C10	1.395 (4)	C30—C29	1.403 (4)
C12—C13	1.374 (4)	C29—C28	1.371 (4)
C12—H20	0.9300	C29—H8	0.9300
C13—C8	1.389 (4)	C28—C27	1.391 (4)
C13—H21	0.9300	C28—H9	0.9300
C8—C9	1.394 (4)	C27—C32	1.387 (4)
C8—N1	1.410 (3)	C27—N3	1.414 (3)
C9—C10	1.370 (4)	C32—C31	1.365 (4)
C9—H23	0.9300	C32—H11	0.9300
C10—H24	0.9300	C31—H12	0.9300
C1—O1	1.348 (3)	C25—C24	1.382 (4)
C1—C2	1.378 (4)	C25—C20	1.403 (4)
C1—C6	1.411 (4)	C25—C26	1.454 (4)
C2—C3	1.380 (4)	C24—C23	1.365 (4)
C2—H26	0.9300	C24—H32	0.9300
C3—C4	1.386 (4)	C23—C22	1.375 (4)
C3—H27	0.9300	C23—H33	0.9300
C4—C5	1.367 (4)	C22—C21	1.377 (4)
C4—H28	0.9300	C22—H34	0.9300
C5—C6	1.388 (4)	C21—C20	1.380 (4)
C5—H29	0.9300	C21—H35	0.9300
C6—C7	1.450 (4)	C20—O2	1.350 (3)
C7—N1	1.278 (3)	C26—N3	1.280 (3)
C7—H37	0.9300	C26—H38	0.9300
N2—H1A	0.8600	N4—H2A	0.8600
O1—H4A	0.8200	O2—H3A	0.8200
			
C16—C15—C14	120.4 (3)	C34—C33—C38	118.6 (3)
C16—C15—H13	119.8	C34—C33—N4	122.6 (3)
C14—C15—H13	119.8	C38—C33—N4	118.7 (3)
C15—C16—C17	121.5 (3)	C33—C38—C37	120.6 (3)
C15—C16—H14	119.3	C33—C38—H2	119.7
C17—C16—H14	119.3	C37—C38—H2	119.7
C18—C17—C16	118.4 (3)	C36—C37—C38	120.3 (3)
C18—C17—H15	120.8	C36—C37—H3	119.9
C16—C17—H15	120.8	C38—C37—H3	119.9
C17—C18—C19	120.9 (3)	C37—C36—C35	119.1 (3)
C17—C18—H16	119.6	C37—C36—H4	120.4
C19—C18—H16	119.6	C35—C36—H4	120.4
C14—C19—C18	120.7 (3)	C34—C35—C36	120.9 (3)
C14—C19—H17	119.7	C34—C35—H5	119.6
C18—C19—H17	119.7	C36—C35—H5	119.6
C15—C14—C19	118.1 (3)	C35—C34—C33	120.5 (3)
C15—C14—N2	123.9 (2)	C35—C34—H6	119.7
C19—C14—N2	117.9 (2)	C33—C34—H6	119.7
C12—C11—N2	119.1 (2)	N4—C30—C31	118.5 (3)
C12—C11—C10	117.9 (3)	N4—C30—C29	123.1 (3)
N2—C11—C10	122.9 (3)	C31—C30—C29	118.3 (3)
C13—C12—C11	121.2 (3)	C28—C29—C30	120.0 (3)
C13—C12—H20	119.4	C28—C29—H8	120.0
C11—C12—H20	119.4	C30—C29—H8	120.0
C12—C13—C8	121.2 (3)	C29—C28—C27	121.6 (3)
C12—C13—H21	119.4	C29—C28—H9	119.2
C8—C13—H21	119.4	C27—C28—H9	119.2
C13—C8—C9	117.7 (2)	C32—C27—C28	117.7 (3)
C13—C8—N1	116.3 (2)	C32—C27—N3	115.9 (2)
C9—C8—N1	126.0 (2)	C28—C27—N3	126.4 (2)
C10—C9—C8	121.1 (3)	C31—C32—C27	121.5 (3)
C10—C9—H23	119.5	C31—C32—H11	119.2
C8—C9—H23	119.5	C27—C32—H11	119.2
C9—C10—C11	121.0 (3)	C32—C31—C30	120.8 (3)
C9—C10—H24	119.5	C32—C31—H12	119.6
C11—C10—H24	119.5	C30—C31—H12	119.6
O1—C1—C2	118.6 (3)	C24—C25—C20	118.7 (2)
O1—C1—C6	121.2 (2)	C24—C25—C26	121.1 (2)
C2—C1—C6	120.1 (3)	C20—C25—C26	120.2 (2)
C1—C2—C3	120.1 (3)	C23—C24—C25	121.7 (3)
C1—C2—H26	120.0	C23—C24—H32	119.2
C3—C2—H26	120.0	C25—C24—H32	119.2
C2—C3—C4	120.4 (3)	C24—C23—C22	119.5 (3)
C2—C3—H27	119.8	C24—C23—H33	120.2
C4—C3—H27	119.8	C22—C23—H33	120.2
C5—C4—C3	119.6 (3)	C21—C22—C23	120.2 (3)
C5—C4—H28	120.2	C21—C22—H34	119.9
C3—C4—H28	120.2	C23—C22—H34	119.9
C4—C5—C6	121.5 (3)	C22—C21—C20	120.7 (3)
C4—C5—H29	119.2	C22—C21—H35	119.7
C6—C5—H29	119.2	C20—C21—H35	119.7
C5—C6—C1	118.2 (2)	O2—C20—C21	119.2 (2)
C5—C6—C7	121.0 (2)	O2—C20—C25	121.6 (2)
C1—C6—C7	120.8 (2)	C21—C20—C25	119.2 (3)
N1—C7—C6	121.4 (2)	N3—C26—C25	121.4 (2)
N1—C7—H37	119.3	N3—C26—H38	119.3
C6—C7—H37	119.3	C25—C26—H38	119.3
C14—N2—C11	126.7 (2)	C30—N4—C33	128.2 (2)
C14—N2—H1A	116.5	C30—N4—H2A	116.0
C11—N2—H1A	116.9	C33—N4—H2A	115.8
C7—N1—C8	124.5 (2)	C26—N3—C27	124.4 (2)
C1—O1—H4A	109.7	C20—O2—H3A	109.8
			
C14—C15—C16—C17	0.9 (4)	C34—C33—C38—C37	−0.8 (4)
C15—C16—C17—C18	−0.1 (5)	N4—C33—C38—C37	−177.0 (3)
C16—C17—C18—C19	−1.2 (5)	C33—C38—C37—C36	−0.1 (5)
C17—C18—C19—C14	1.8 (5)	C38—C37—C36—C35	1.0 (5)
C16—C15—C14—C19	−0.2 (4)	C37—C36—C35—C34	−0.9 (5)
C16—C15—C14—N2	−177.0 (3)	C36—C35—C34—C33	0.0 (5)
C18—C19—C14—C15	−1.1 (4)	C38—C33—C34—C35	0.9 (4)
C18—C19—C14—N2	175.8 (3)	N4—C33—C34—C35	176.9 (3)
N2—C11—C12—C13	176.7 (3)	N4—C30—C29—C28	176.2 (3)
C10—C11—C12—C13	0.4 (4)	C31—C30—C29—C28	0.2 (4)
C11—C12—C13—C8	−0.2 (4)	C30—C29—C28—C27	−0.5 (4)
C12—C13—C8—C9	0.6 (4)	C29—C28—C27—C32	0.0 (4)
C12—C13—C8—N1	179.4 (3)	C29—C28—C27—N3	−179.6 (3)
C13—C8—C9—C10	−1.3 (4)	C28—C27—C32—C31	0.6 (4)
N1—C8—C9—C10	−179.9 (3)	N3—C27—C32—C31	−179.7 (3)
C8—C9—C10—C11	1.5 (4)	C27—C32—C31—C30	−0.9 (4)
C12—C11—C10—C9	−1.1 (4)	N4—C30—C31—C32	−175.8 (3)
N2—C11—C10—C9	−177.2 (3)	C29—C30—C31—C32	0.4 (4)
O1—C1—C2—C3	178.5 (2)	C20—C25—C24—C23	−0.4 (4)
C6—C1—C2—C3	−2.7 (4)	C26—C25—C24—C23	179.2 (2)
C1—C2—C3—C4	1.2 (4)	C25—C24—C23—C22	1.2 (5)
C2—C3—C4—C5	−0.2 (5)	C24—C23—C22—C21	−1.0 (4)
C3—C4—C5—C6	0.8 (5)	C23—C22—C21—C20	0.1 (4)
C4—C5—C6—C1	−2.3 (4)	C22—C21—C20—O2	−179.2 (3)
C4—C5—C6—C7	175.9 (3)	C22—C21—C20—C25	0.7 (4)
O1—C1—C6—C5	−178.0 (2)	C24—C25—C20—O2	179.3 (3)
C2—C1—C6—C5	3.2 (4)	C26—C25—C20—O2	−0.3 (4)
O1—C1—C6—C7	3.7 (4)	C24—C25—C20—C21	−0.5 (4)
C2—C1—C6—C7	−175.0 (2)	C26—C25—C20—C21	179.9 (3)
C5—C6—C7—N1	−178.5 (2)	C24—C25—C26—N3	−179.7 (3)
C1—C6—C7—N1	−0.4 (4)	C20—C25—C26—N3	−0.1 (4)
C15—C14—N2—C11	−4.5 (4)	C31—C30—N4—C33	−168.0 (3)
C19—C14—N2—C11	178.8 (3)	C29—C30—N4—C33	16.0 (5)
C12—C11—N2—C14	134.9 (3)	C34—C33—N4—C30	44.6 (5)
C10—C11—N2—C14	−49.0 (4)	C38—C33—N4—C30	−139.4 (3)
C6—C7—N1—C8	178.8 (2)	C25—C26—N3—C27	178.5 (2)
C13—C8—N1—C7	−179.3 (2)	C32—C27—N3—C26	173.9 (3)
C9—C8—N1—C7	−0.7 (4)	C28—C27—N3—C26	−6.4 (4)

### Hydrogen-bond geometry (Å, º)

Cg1 is the centroid of ring C1–C6 in molecule *A*.

**Table:**

*D*—H···*A*	*D*—H	H···*A*	*D*···*A*	*D*—H···*A*
O1—H4*A*···N1	0.82	1.86	2.568 (3)	144
O2—H3*A*···N3	0.82	1.82	2.550 (3)	148
N2—H1*A*···O2^i^	0.86	2.29	3.006 (4)	141
N4—H2*A*···O1^ii^	0.86	2.33	3.179 (4)	168
C15—H13···*Cg*1^iii^	0.93	2.92	3.581 (4)	129

Symmetry codes: (i) *x*, *y*, *z*−1; (ii) *x*, *y*+1, *z*+1; (iii) −*x*, *y*+1/2, −*z*+1.

## References

[bb1] Altomare, A., Burla, M. C., Camalli, M., Cascarano, G. L., Giacovazzo, C., Guagliardi, A., Moliterni, A. G. G., Polidori, G. & Spagna, R. (1999). *J. Appl. Cryst.* **32**, 115–119.

[bb2] Brandenberg, K. & Putz, H. (2006). *DIAMOND*. Crystal Impact GbR, Bonn, Germany.

[bb3] Bruker (2003). *SMART* and *SAINT*. Bruker AXS Inc., Madison, Wisconsin, USA.

[bb4] Dalapati, S., Alam, M. A., Jana, S. & Guchhait, N. (2011). *J. Fluor. Chem.* **132**, 536–540.

[bb5] Faizi, M. S. H. & Hussain, S. (2014). *Acta Cryst.* E**70**, m197. 10.1107/S160053681400957X PMC4051090 24940192

[bb6] Faizi, M. S. H. & Sen, P. (2014). *Acta Cryst.* E**70**, m173. 10.1107/S1600536814007077 PMC4011313 24860307

[bb7] Groom, C. R. & Allen, F. H. (2014). *Angew. Chem. Int. Ed.* **53**, 662–671. 10.1002/anie.201306438 24382699

[bb8] Lozier, R. H., Bogomolni, R. A. & Stoeckenius, W. (1975). *Biophys. J.* **15**, 955–962. 10.1016/S0006-3495(75)85875-9 PMC1334761 1182271

[bb9] Macrae, C. F., Bruno, I. J., Chisholm, J. A., Edgington, P. R., McCabe, P., Pidcock, E., Rodriguez-Monge, L., Taylor, R., van de Streek, J. & Wood, P. A. (2008). *J. Appl. Cryst.* **41**, 466–470.

[bb10] Moroz, Y. S., Demeshko, S., Haukka, M., Mokhir, A., Mitra, U., Stocker, M., Müller, P., Meyer, F. & Fritsky, I. O. (2012). *Inorg. Chem.* **51**, 7445–7447. 10.1021/ic300902z 22765646

[bb11] Nor Hashim, N. Z., Kassim, K. & Yamin, B. M. (2010). *Acta Cryst.* E**66**, o2039. 10.1107/S160053681002742X PMC3007474 21588348

[bb12] Penkova, L., Demeshko, S., Pavlenko, V. A., Dechert, S., Meyer, F. & Fritsky, I. O. (2010). *Inorg. Chim. Acta*, **363**, 3036–3040.

[bb13] Petrusenko, S. R., Kokozay, V. N. & Fritsky, I. O. (1997). *Polyhedron*, **16**, 267–274.

[bb14] Safin, D. A., Robeyns, K. & Garcia, Y. (2012). *RSC Adv.* **2**, 11379–11388.

[bb15] Sheldrick, G. M. (2004). *SADABS*. University of Göttingen, Germany.

[bb16] Sheldrick, G. M. (2008). *Acta Cryst.* A**64**, 112–122. 10.1107/S0108767307043930 18156677

[bb17] Sliva, T. Yu., Duda, A. M., Głowiak, T., Fritsky, I. O., Amirkhanov, V. M., Mokhir, A. A. & Kozłowski, H. (1997). *J. Chem. Soc. Dalton Trans.* pp. 273–276.

[bb18] Spek, A. L. (2009). *Acta Cryst.* D**65**, 148–155. 10.1107/S090744490804362X PMC2631630 19171970

[bb19] Sun, Y., Wang, Y., Liu, Z., Huang, C. & Yu, C. (2012). *Spectrochim. Acta Part A*, **96**, 42–50. 10.1016/j.saa.2012.04.094 22652542

[bb20] Xie, Y.-Z., Shan, G.-G., Li, P., Zhou, Z.-Y. & Su, Z.-M. (2013). *Dyes and Pigments*, **96**, 467–474.

